# Complete genome sequence of the novel virulent phage PMBT24 infecting *Enterocloster bolteae* from the human gut

**DOI:** 10.1016/j.heliyon.2024.e28813

**Published:** 2024-04-05

**Authors:** Sabrina Sprotte, Erik Brinks, Horst Neve, Charles M.A.P. Franz

**Affiliations:** Department of Microbiology and Biotechnology, Max Rubner-Institut, Federal Research Institute of Nutrition and Food, Hermann-Weigmann-Str. 1, 24103, Kiel, Germany

**Keywords:** Novel phage PMBT24, *Enterocloster bolteae*, Complete genome, Human gut

## Abstract

PMBT24, the first reported virulent bacteriophage infecting the anaerobic human gut bacterium *Enterocloster bolteae* strain MBT-21, was isolated from a municipal sewage sample and its genome was sequenced and analysed. Transmission electron microscopy revealed a phage with an icosahedral head and a long, non-contractile tail. The circularly permutated, 99,962-bp dsDNA genome of the *pac-*type phage has a mol% G + C content of 32.1 and comprises 173 putative ORFs. Using amino acid sequence-based phylogeny, phage PMBT24 showed similarity to other, hitherto non-published phage genomes in the databases. Our data suggested phage PMBT24 to present the type phage of a novel genus (proposed name Kielvirus) and novel family of phages (proposed name Kielviridae).

## 1 Introduction

*Enterocloster bolteae* [1] (formerly *Clostridium bolteae* [1,2]) is an opportunistic pathogen that occurs in the human gut. This species is obligately anaerobic and capable of forming spores [2]. It was found to be present with increased abundance in the intestines of autistic children [[3], [4], [5], [6]] and was also associated with bacteremia, intra-abdominal infections and abscesses [5]. Frequently described antibiotic resistances of *E. bolteae* strains include ampicillin, erythromycin, lincomycin, ciprofloxacin, doxycycline and colistin [3]. Because of these multidrug resistance phenotypes, a supportive or alternative treatment for infections with *E. bolteae* besides treatment with antibiotics would be a desirable development. Bacteriophages (phages) are among the most promising alternative tools to combat bacterial infections. They are gaining increasing recognition as potential modulators of the gut ecosystem due to their ability to affect bacterial communities [7,8], e.g. by fecal microbial transplantation, as was previously investigated for *Clostridium difficile* [9,10]. So far, only six complete genome sequences of phages infecting *E. bolteae* have been deposited in the NCBI genome database. In this study, a novel virulent phage PMBT24 infecting *E. bolteae* strain MBT-21 was characterized and its genome was analysed to determine the phage's potential for use as a possible biotherapy tool for controlling infections with this opportunistic pathogen.

## 2 Materials and methods

Phage isolation under anaerobic conditions were performed out in a Whitley A45 anaerobic workstation (Meintrup DWS Laborgeräte GmbH, Herzlake, Germany) maintained with an anaerobic gas mixture (ANO2: 10% hydrogen, 10% carbon dioxide and 80% nitrogen) was performed as described previously, using dehydrated Anaerobe Basal Broth (ABB; Thermo Fisher Scientific) and sewage samples from a municipal sewage treatment plant near to Kiel, Germany [11]. The host bacterium *E. bolteae* MBT-21 was isolated from human feces and cultivated in ABB under anaerobic conditions. The phage lysate was concentrated from the supernatant of 1 L of a phage PMBT24 infected *E. bolteae* MBT-21 culture using cesium chloride (CsCl) density gradient ultracentrifugation [12] and was subsequently analysed by transmission electron microscopy (TEM), as described elsewhere [11].

For genome sequencing, the bacterial DNA was prepared using the Quick-DNA™ Fungal/Bacterial MiniPrep Kit (ZymoResearch, Freiburg, Germany) and the genome was sequenced using the MiSeq Reagent Nano Kit v2 (Illumina, Munich, Germany) according to the manufacturers’ instructions and sequencing was performed on a MiSeq sequencer (Illumina, Munich, Germany). The MiSeq® Reporter software was used for base calling, demultiplexing and adapter trimming directly on the MiSeq sequencer. The reads were *de novo* assembled using SPAdes version 3.10.0 (Bacterial and Viral Bioinformatics Resource Center, BV-BRC) [13]. Automatic annotation of the bacterial genome was performed with RASTtk (BV-BRC) [14,15] and the bacterium was identified by BLASTN comparison to bacterial genome sequences in the GenBank database.

Phage DNA was isolated from high titer phage lysate obtained from CsCl density gradient using the same kit with some previously described modifications [11]. For phage genome sequencing, the TruSeq DNA Library Preparation Kit was used according to the manufacturers’ instructions. Sequencing and quality control of the phage genome was performed as described before. The reads were *de novo* assembled using Unicycler v0.4.8 (BV-BRC) [15]. Annotation of the phage genome was performed automatically with VIGOR4 in bacteriophage mode (BV-BRC) [14,15], followed by manual comparison of predicted proteins with proteins in the databases using BLASTP [16] and HHPred [17]. BLASTP results were only accepted with E-values less than 1 × 10^−5^, with a query coverage of ≥60% and an identity of ≥35%. Using HHPred, proteins were compared to the PDB, UniProt, Pfam and SMART databases (accessed on 15 March 2023). Results were only accepted with E-values less than 1 × 10^−3^ and a minimum probability in the hitlist of 20%. Putative host and phage promoters in intergenic regions were identified using PhagePromoter [18] and putative rho-independent terminators occurring in intergenic regions were determined using ARNold [19], which computes the free energy of the predicted terminator stem-loops. For identification of phage termini and analysis of the DNA packaging mechanism, PhageTerm 1.0.12 [20] was used. The phage genome was analysed for the presence of tRNA genes with tRNAscan-SE version 2.0 [21,22]. The PHACTS software (Galaxy Version 0.3) [23] was applied to predict the phage lifestyle (i.e., whether lysogenic or lytic). ResFinder 4.1 [20,[24], [25], [26], [27]] was used for the prediction of antibiotic resistance genes and VirulenceFinder 2.0 [28,29] for the prediction of bacterial virulence genes on the phage genome. The phage genome map was generated using Geneious version 9.1.8.

A comparison of the phage PMBT24 genome to other phage genomes deposited in the databases of the National Center for Biotechnology Information (NCBI) and the European Nucleotide Archive (ENA) was performed at the nucleotide level with megablast on the BLASTN platform [16] (accessed on 2 May 2023). The resulting identity and coverage values were used to calculate the sequence similarity according to Turner et al. [30]. The identity between phage PMBT24 and CB457P1 (acc. no. OP172753.1) at the amino acid level was plotted according to tBLASTx using the genome comparison visualiser Easy Fig 2.2.3 [31]. In addition, the phage PMBT24 tape measure protein was compared to those of the closest related phages CB457P1 and cteZU1 (acc. no. BK021559.1) using BLASTP. The Virus Classification and Tree Building Online Resource (VICTOR) was used for a whole amino acid sequence-based phylogeny and classification. In this analysis all pairwise comparisons of the amino acid sequences of phage PMBT24 and of the 22 closest related phages from the megablast analysis were conducted using the Genome-BLAST Distance Phylogeny (GBDP) method [32], with settings recommended for prokaryotic viruses [33]. The evolution tree was reconstructed with the formula D6, rooted at the midpoint [34] and visualised with ggtree [35]. Taxon boundaries at the species, genus and family level were estimated automatically by VICTOR [33,36,37]. A list of the analysed phages is shown in Supplementary Table S1. In addition, VIRIDIC (Virus Intergenomic Distance Calculator) [38] was used to calculate the pairwise intergenomic similarities amongst these phages. It uses the traditional algorithm used by the International Committee on Taxonomy of Viruses (ICTV), Bacterial and Archaeal Viruses Subcommittee for calculation. All phage genomes were also used as queries to build a whole-genome tree with the Viral Proteomic Tree server (ViPTree) version 3.6 [39]. ViPTree calculates the sequence similarities of the whole genomes using tBLASTx. Furthermore, the amino acid sequence of the putative terminase large subunit from phage PMBT24 was compared in coverage and identity to the most closely related proteins using BLASTP.

## 3 Results

**Fig. 1:**
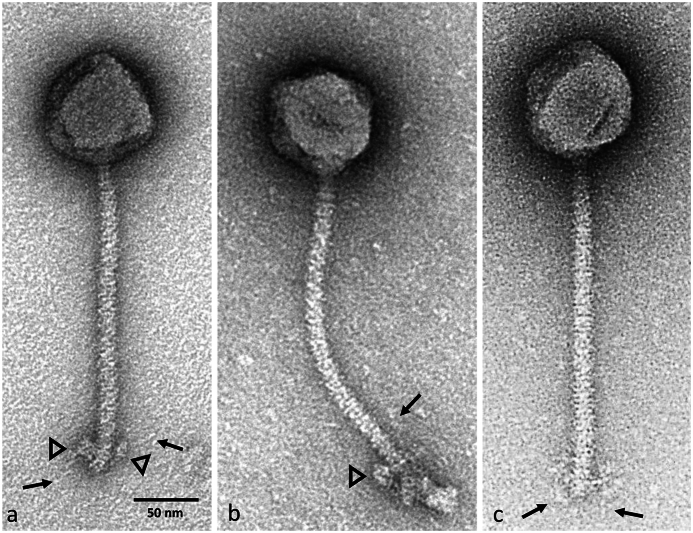
Electron micrographs of *E. bolteae* phage PMBT24 stained with 1% (w/v) uranyl acetate. The PMBT24 tail is coated by thin spiral structures. Globular structures at the distal tail end are indicated by the triangles (a–b). The arrows indicate thin tail fibers visible at the tail tip (a–c).

Electron micrographs of the CsCl gradient purified phage PMBT24 revealed a siphovirus morphotype with an icosahedral head of 84 ± 1.3 nm (n = 7) in diameter and a 238 ± 6.5 nm (n = 7) long, flexible and non-contractile tail (Fig. 1). Globular appendages were visible at the distal tail ends and thin tail fibers at the tail tip. Regarding plaque formation, phage PMBT24 formed small turbid plaques with a diameter of 0.5–1 mm on an *E. bolteae* MBT-21 lawn (data not shown).

**Fig. 2:**
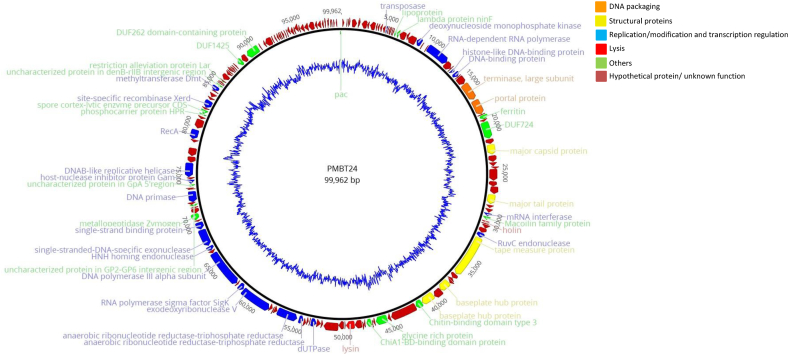
Circular map of the phage PMBT24 genome with structural and functional annotations and mol% GC content (inner circle) created by Geneious. Arrows indicate the predicted ORFs, which are labeled and color-coded based on their predicted function (see colour legend for details). The *pac*-site is shown in green at the top of the map. (For interpretation of the references to colour in this figure legend, the reader is referred to the Web version of this article.)

All raw reads from the host bacterium *E. bolteae* MBT-21 were deposited in the NCBI SRA with accession number PRJNA1078606. Phage genome sequencing generated 258,014 paired-end reads (2 × 251 bp). The mean read length averaged 235 bp. The reads were *de novo* assembled into a single contig with a total length of 99,962 bp and a mol% G + C content of 32.1. The assembled genome showed a high 416-fold coverage. Amongst the predicted 173 open reading frames (ORFs), 48 ORFs (28%) could be assigned a putative function, while 125 ORFs (72%) encoding hypothetical proteins were unclassified (Supplementary Table S2). The ORFs for which a putative function could be assigned were categorized into five functional groups (Fig. 2): *i) DNA packaging:* large terminase subunit (ORF36), portal protein (ORF37); *ii) structural proteins:* major capsid protein (ORF43), major tail protein (ORF50), tape measure protein (ORF60), baseplate hub proteins (ORF64, ORF66); *iii) DNA replication/modification and transcription regulation:* dUTPase (ORF83), ribonucleotide reductase (anaerobic) (ORF87, ORF88), exodeoxyribonuclease V (ORF92), RNA polymerase sigma factor SigK (ORF93), DNA polymerase III alpha subunit (ORF95), HNH homing endonuclease (ORF97), single-stranded-DNA-specific exonuclease (ORF98), DNA primase (ORF106), host-nuclease inhibitor protein Gam (ORF110), DNAB-like replicative helicase (ORF112), RecA (ORF116), site-specific recombinase Xerd (ORF122), methyltransferase Dmt (ORF126), restriction alleviation protein Lar (ORF130); *iv) host cell lysis:* holin (ORF55), lysin (ORF77) and *v) other functions:* e.g., lipoprotein (ORF21), lambda protein ninF (ORF22) and ferritin (ORF39). 97 ORFs were located on the positive and 76 ORFs were located on the negative strand. Putative host and phage promoters and putative *rho*-independent terminators occurring in intergenic regions were identified (both Supplementary Tables S3 and S4). According to the PhageTerm Method, phage PMBT24 contains a circularly permutated genome with redundant ends used to circularize the genome. As a significant peak with τ > 0.1 was found only on the reverse strand, the phage was considered a *pac*-type phage, which uses the headful DNA packaging mechanism initiated at the *pac* site. The *pac* site was selected as the start of the phage PMBT24 genome map (Fig. 2). No tRNAs could be identified in the phage genome. Phage lifestyle was predicted to be strictly lytic. Further analysis revealed the absence of antibiotic resistance and bacterial virulence genes in the genome of phage PMBT24.

**Fig. 3:**
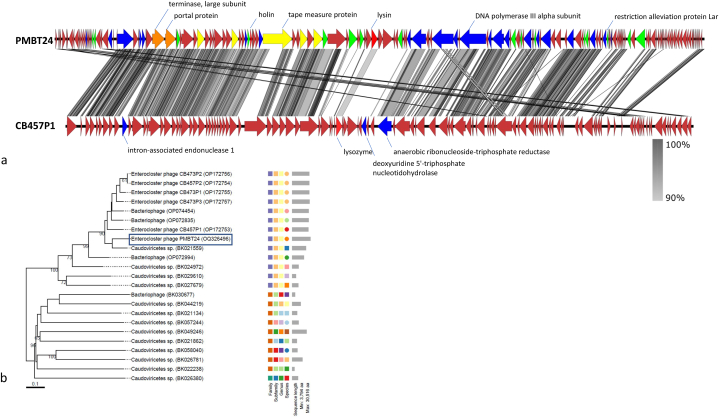
a. Alignment of phages PMBT24 and CB457P1 genomes at the amino acid level created by Easyfig. b. Phylogenomic analysis of phage PMBT24 with the 22 closest related phages from the NCBI database at the amino acid level using VICTOR. The tree is based on the recommended formula D6 and shows GBDP pseudo-bootstrap support values from 100 replications above branches.

With a coverage of 77% and an identity of 96.55%, the assembled genome showed closest similarity (i.e., 74.34% calculated by % identity multiplied by % coverage according to Turner et al. [30]) to the genome of *E*. *bolteae* phage CB457P1, which has a slightly smaller size of 96,610 bp. Furthermore, the phage PMBT24 genome showed 67.41% similarity to the partial 72,822 bp-genome fragment of a *Caudoviricetes* sp. isolate cteZU1 from a metagenome assembled genome [40]. Genome comparison at the amino acid level of phages PMBT24 and CB457P1 showed a similar genome organisation (Fig. 3a). More precisely, the genomes showed a higher identityin the 5′ end where the packaging proteins and the structural protein genes are located than in the middle region and 3′end where genes for replication, modification and transcription regulation proteins are located. The predicted structural proteins, i.e. the major capsid protein, major tail protein and baseplate hub protein I and II showed 90–95% identity between the two phages. In contrast, the tape measure proteins were of different organization. Thus, homologous sequences of the PMBT24 tape measure protein's (1508 aa) C- and N-termini were found in two different ORFs (ORF33 with 388 aa and ORF35 with 672 aa) on the genome of phage CB457P1, and these are separated by ORF34 (157 aa) which shows no similarity to any sequence found in PMBT24. A 1511 aa protein with 78.40% sequence identity to the tape measure protein of phage PMBT24 was furthermore identified in the closely related phage cteZU1 using BLASTP (data not shown).

**Fig. 4:**
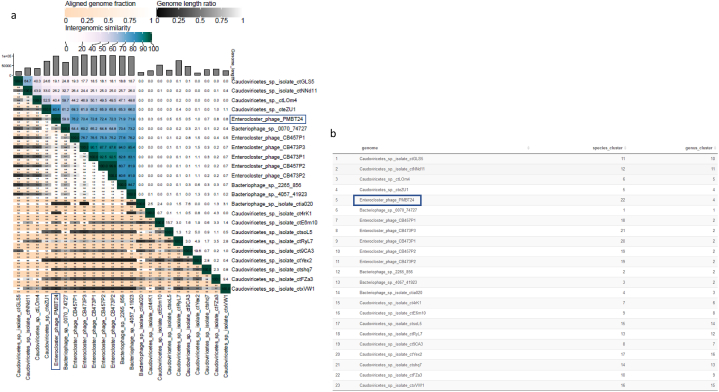
VIRIDIC heatmap of phage PMBT24 and its 22 closest related phages (a) and corresponding species and genus cluster (b).

**Fig. 5:**
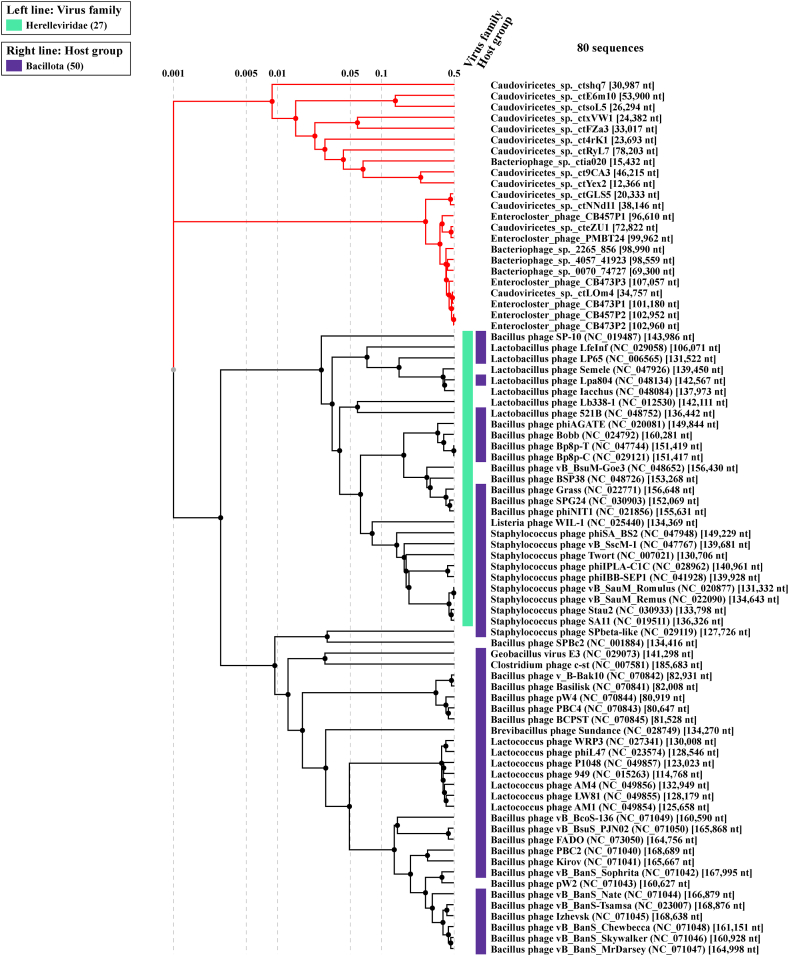
ViPTree showing phylogenetic relationship of phage PMBT24 and its 22 closest related phages used as queries (marked with asterisks). The rectangular tree only shows related genomes (sequence similarities SG > 0.02).

Analysis of phage PMBT24 and its 22 closest related phages from the megablast analysis using VICTOR suggested that phage PMBT24 represents a new species in a yet unassigned genus together with 12 other phages (Fig. 3b). The VIRIDIC heatmap showed 80.4% and 76.2% intergenomic similarity between phage PMBT24 and cteZU1 (partial genome) and CB457P1, respectively, and PMBT24 belongs to the same unassigned genus as phage cteZU1. In contrast to the VICTOR analysis, phage PMBT24 and cteZU1 were assigned to another genus cluster as the remaining 21 phages (Fig. 4). However, the analysis with VIRIDIC revealed that two phages (CB457P2 and CB473P2) within this group belong to the same species, while the analysis with VICTOR revealed four phages (CB473P1, CB473P2, CB473P3, CB457P2). In a ViPTree, which only showed related phage genomes with sequence similarities S_G_ > 0.02, phage PMBT24 and 12 of the 22 analysed phages were connected by deep branches between 0.1 and 0.5 (Fig. 5). They formed a distinct cluster near the *Herelleviridae* phage family, which includes *Bacillus*, *Lactobacillus*, *Staphylococcus* and *Listeria* phages. The remaining 10 analysed phages clustered elsewhere. A comparison of the predicted terminase large subunit protein of PMBT24 with that from the 10 closest related *Enterocloster* phages showed that these proteins of phage PMBT24 and cteZU1 were identical (100% coverage and identity), while the corresponding proteins of PMBT24 and CB457P1 and the remaining proteins showed only low identity (data not shown).

## 4 Discussion

So far, only six complete genome sequences of *E*. *bolteae* phages (acc. no. OP172753, OP172756, OP172754, OP172755, OP172757, OP074454) and several partial genomes stemming from metagenomic projects showing similarity to *Enterocloster* phages have been deposited in the NCBI genome database (accessed on 27 November 2023). However, detailed characterizations of these phages have not been published so far. The bacterium *E. bolteae* has been linked to autism, bacteremia, intra-abdominal infections and abscesses and some strains also exhibit antibiotic resistance. In this study, phage PMBT24 has been isolated under anaerobic conditions from sewage and was shown to infect the *E. bolteae* MBT-21 isolate from the human gut. The phage displayed a siphovirus morphotype with an icosahedral head and a long, non-contractile tail. Globular appendages are visible at the distal tail ends, which are also known to occur in other tailed phages [41]. Notably, the whole tail surface is covered by thin spiral structures originally described for *Lactococcus lactis* Skunavirus phages [42,43].

Using the formula according to Turner et al. [30], who proposed that a phage represents the first isolate of a new species in an undefined or existing genus if its genome exhibits <95% but ≥70% sequence similarity (calculated by % identity multiplied by % coverage) at the nucleotide level over the full genome length to other phages in the databases, phage PMBT24 showed sequence similarity under the 95%-threshold to the closest related phages CB457P1 and cteZU1, suggesting that phage PMBT24 represents a new phage species. However, a comparison with phage cteZU1 was difficult, because only the partial genome is available for this phage. Therefore, the genome of phage PMBT24 was compared to the complete genome of the next closely related phage CB457P1 at the amino acid level using EasyFig. Interestingly, despite high similarity between the structural proteins, i.e. major capsid protein, major tail protein and baseplate hub protein I and II, there is only low identity between the tape measure protein of phage PMBT24 and the corresponding protein(s) in the genome of phage CB457P1. The tail length of a phage is proportional to the length of the tape measure protein corresponding gene [44]. This could be an indication that the two phages differ in tail length.

Comparative analysis from VICTOR and VIRIDIC revealed that phage PMBT24 belongs to an unassigned genus together with phage cteZU1 (according to VIRIDIC) or 11 further closely related phages besides phage cteZU1 (according to VICTOR). However, the analysis revealed that at least two of these phages belong to the same species. As VIRIDIC builds on and improves the traditional BLASTN method used by Bacterial and Archaeal Viruses Subcommittee from ICTV to calculate and visualize virus intergenomic relatedness, these results should be more reliable [38]. In addition, a comparative analysis of the predicted terminase large subunit protein could confirm the hypothesis that phage PMBT24 and cteZU1 belong to the same unassigned genus, as the proteins showed 100% coverage and 99.84% aa identity (data not shown). All other terminase large subunit proteins used for the analysis showed no significant similarity. The terminase large subunit is considered the most universally conserved gene sequence in phages and therefore is often used to construct phylogeny [45,46].

Finally, the deep branch lengths compared to related phages observed in the ViPTree suggested that the same 13 phages including phage PMBT24 furthermore represent a new unassigned family near the *Herelleviridae* phage family. However, phylogenetic analysis of the phages was difficult because the most deposited genome sequences were partial genomes. To formally classify these phages, we propose to create the new bacteriophage family Kielviridae which includes the novel Kielvirus genus with PMBT24 as the type phage. Phage PMBT24 has the potential to be used as biotherapy tool for controlling infections with the opportunistic pathogen *E. bolteae*, although physiological parameters, e.g. latent period, burst size, pH and temperature stability as well as host range, still need to be investigated in a further study. However, some of these parameters are currently challenging to investigate, because of the need for culturing of the *E. bolteae* host bacterium under anaerobic conditions. Our study presents new data on phage genome sequence of the first described *E. bolteae* phage and increases current knowledge on *Enterocloster* phages, which may aid in future investigations on the phylogeny of these phages. To our knowledge, this is the first report of a virulent bacteriophage infecting *E. bolteae*.

## Ethics statement

This study was approved by the Ethics Committee of the Faculty of Medicine, Kiel University, Germany with the ethics approval reference D420/20 on February 7, 2020. Written informed consent was obtained from all the participants.

## Data availability statement

The complete genome sequence of phage PMBT24 generated in this project was deposited in the NCBI database under the GenBank accession number OQ326496.2. All raw reads from the host bacterium *E. bolteae* MBT-21 were deposited in the NCBI SRA with accession number PRJNA1078606.

## CRediT authorship contribution statement

**Sabrina Sprotte:** Writing – original draft. **Erik Brinks:** Writing – review & editing. **Horst Neve:** Writing – review & editing. **Charles M.A.P. Franz:** Supervision.

## Declaration of competing interest

The authors declare that they have no known competing financial interests or personal relationships that could have appeared to influence the work reported in this paper.
