# Supplementary material for: Complete genome sequence of the novel virulent phage PMBT24 infecting Enterocloster bolteae from the human gut
Source: Heliyon. 2024 Apr 5;10(8):e28813. doi: 10.1016/j.heliyon.2024.e28813 (PMC11035940; doi:10.1016/j.heliyon.2024.e28813)
Supplement: Multimedia component 1 [file mmc1.docx]

|  | Definition at NCBI | Genome size | Genome completeness | Accession no. |
| --- | --- | --- | --- | --- |
| 1 | Enterocloster phage PMBT24 | 99962 nt | complete | OQ326496.2 |
| 2 | Caudoviricetes sp. isolate cteZU1 | 72822 nt | partial | BK021559.1 |
| 3 | Enterocloster phage CB457P1 | 96610 nt | complete | OP172753.1 |
| 4 | Bacteriophage sp. isolate 0070 74727 | 69300 nt | partial | OP072994.1 |
| 5 | Bacteriophage sp. isolate 4057 41923 | 98559 nt | partial | OP072835.1 |
| 6 | Bacteriophage sp. isolate 2265 856 | 98990 nt | complete | OP074454.1 |
| 7 | Enterocloster phage CB473P2 | 102960 nt | complete | OP172756.1 |
| 8 | Enterocloster phage CB457P2 | 102952 nt | complete | OP172754.1 |
| 9 | Enterocloster phage CB473P1 | 101180 nt | complete | OP172755.1 |
| 10 | Enterocloster phage CB473P3 | 107057 nt | complete | OP172757.1 |
| 11 | Caudoviricetes sp. isolate ctLOm4 | 34757 nt | partial | BK024972.1 |
| 12 | Caudoviricetes sp. isolate ctNNd11 | 38146 nt | partial | BK027679.1 |
| 13 | Caudoviricetes sp. isolate ctGLS5 | 20333 nt | partial | BK029610.1 |
| 14 | Caudoviricetes sp. isolate ctFZa3 | 33017 nt | partial | BK057244.1 |
| 15 | Caudoviricetes sp. isolate ctYex2 | 12366 nt | partial | BK022238.1 |
| 16 | Caudoviricetes sp. isolate ctxVW1 | 24382 nt | partial | BK021134.1 |
| 17 | Caudoviricetes sp. isolate ctshq7 | 30987 nt | partial | BK026380.1 |
| 18 | Caudoviricetes sp. isolate ctRyL7 | 78203 nt | partial | BK049246.1 |
| 19 | Caudoviricetes sp. isolate ctsoL5 | 26294 nt | partial | BK058040.1 |
| 20 | Caudoviricetes sp. isolate ctE6m10 | 53900 nt | partial | BK026781.1 |
| 21 | Bacteriophage sp. isolate ctia020 | 15432 nt | partial | BK030677.1 |
| 22 | Caudoviricetes sp. isolate ct9CA3 | 46215 nt | partial | BK044219.1 |
| 23 | Caudoviricetes sp. isolate ct4rK1 | 23693 nt | partial | BK021862.1 |

**Supplementary Table S1.** Phage genomes (complete and partial) from the NCBI database used for phylogenetic analysis in this study.
