# Supplementary material for: Complete genome sequence of the novel virulent phage PMBT24 infecting Enterocloster bolteae from the human gut
Source: Heliyon. 2024 Apr 5;10(8):e28813. doi: 10.1016/j.heliyon.2024.e28813 (PMC11035940; doi:10.1016/j.heliyon.2024.e28813)
Supplement: Multimedia component 2 [file mmc2.docx]

**Supplementary Table S2.** Overview of the 173 putative ORFs in the genome of *E. bolteae* phage PMBT24 with their predicted function and the best match result in the databases using BlastP and HHpred with significant probability hits.

| **ORF** | **Predicted function** | **Strand** | **Position** | | **Length (bp)** | **Best match BlastP** | **E-Value** | **Ident.(%)** | | **Best match HHPred** | | **structural/domain database** | **Probability (%)** | **E-Value** |  |
| --- | --- | --- | --- | --- | --- | --- | --- | --- | --- | --- | --- | --- | --- | --- | --- |
| 1 | hypothetical protein | + | 158 | 364 | 207 | - | - | | - | | - | - | - | - | |
| 2 | hypothetical protein | + | 608 | 703 | 96 | - | - | | - | | - | - | - | - | |
| 3 | hypothetical protein | + | 772 | 888 | 117 | - | - | | - | | - | - | - | - | |
| 4 | hypothetical protein | + | 885 | 998 | 114 | - | - | |  | | - | - | - | - | |
| 5 | hypothetical protein | + | 1,185 | 1,715 | 531 | hypothetical protein [Enterocloster] | 1e-125 | | 100 | | - | - | - | - | |
| 6 | hypothetical protein | + | 1,838 | 1,933 | 96 | - | - | | - | | - | - | - | - | |
| 7 | hypothetical protein | + | 1,908 | 2,057 | 150 | - | - | | - | | - | - | - | - | |
| 8 | hypothetical protein | - | 2,180 | 2,761 | 582 | - | - | | - | | - | - | - | - | |
| 9 | hypothetical protein | - | 2,773 | 3,018 | 246 | hypothetical protein [Bacteriophage sp.] | 1e-38 | | 79.27 | | - | - | - | - | |
| 10 | hypothetical protein | - | 3,050 | 3,667 | 618 | hypothetical protein CB457P1_00103 [Enterocloster phage CB457P1] | 1e-108 | | 82.81 | | - | - | - | - | |
| 11 | hypothetical protein | - | 3,685 | 3,867 | 183 | hypothetical protein CB457P1_00104 [Enterocloster phage CB457P1] | 4e-33 | | 90 | | - | - | - | - | |
| 12 | hypothetical protein | - | 3,882 | 4,118 | 237 | hypothetical protein CB457P2_00125 [Enterocloster phage CB457P2] | 8e-41 | | 82.05 | | - | - | - | - | |
| 13 | hypothetical protein | - | 4,136 | 4,420 | 285 | hypothetical protein CB457P2_00126 [Enterocloster phage CB457P2] | 2e-58 | | 95.74 | | - | - | - | - | |
| 14 | hypothetical protein | - | 4,439 | 4,723 | 285 | hypothetical protein [Bacteriophage sp.] | 2e-37 | | 82.14 | | - | - | - | - | |
| 15 | transposase | - | 4,727 | 4,954 | 228 | transposase IS200 like [Bacteriophage sp.] | 2e-29 | | 84.21 | | - | - | - | - | |
| 16 | hypothetical protein | - | 4,980 | 5,105 | 126 | - | - | | - | | - | - | - | - | |
| 17 | hypothetical protein | - | 5,115 | 5,279 | 165 | - | - | | - | | - | - | - | - | |
| 18 | hypothetical protein | + | 5,281 | 5,391 | 111 | - | - | | - | | - | - | - | - | |
| 19 | hypothetical protein | + | 5,490 | 5,591 | 102 | - | - | | - | | - | - | - | - | |
| 20 | hypothetical protein | + | 5,595 | 5,699 | 105 | - | - | | - | | - | - | - | - | |
| 21 | lipoprotein | - | 5,705 | 5,947 | 243 | lipoprotein [Enterocloster] | 7e-39 | | 95.71 | | - | - | - | - | |
| 22 | lambda protein ninF | - | 5,996 | 6,307 | 312 | zinc ribbon domain-containing protein [Lacrimispora amygdalina] | 4e-07 | | 36.67 | | NINF_LAMBD Protein ninF [Escherichia phage lambda] | Uniprot: P03769 | 98.13 | 0.0000017 | |
| 23 | hypothetical protein | - | 6,313 | 7,035 | 723 | Thymidylate synthase complementing protein [Bacteriophage sp.] | 1e-137 | | 76.89 | | - | - | - | - | |
| 24 | hypothetical protein | - | 7,035 | 7,292 | 258 | - | - | | - | | - | - | - | - | |
| 25 | hypothetical protein | - | 7,285 | 8,208 | 924 | hypothetical protein CB457P1_00116 [Enterocloster phage CB457P1] | 0.0 | | 98.36 | | - | - | - | - | |
| 26 | deoxynucleoside monophosphate kinase | - | 8,201 | 8,797 | 597 | hypothetical protein CB457P1_00117 [Enterocloster phage CB457P1] | 8e-121 | | 100 | | deoxynucleoside monophosphate kinase [Enterobacteria phage T4] | PDB: 1DEK_B | 99.6 | 2.7e-13 | |
| 27 | hypothetical protein | - | 8,973 | 9,074 | 102 | - | - | | - | | - | - | - | - | |
| 28 | hypothetical protein | - | 9,507 | 9,617 | 111 | - | - | | - | | - | - | - | - | |
| 29 | RNA-dependent RNA polymerase | + | 9,612 | 12,107 | 2,496 | RNA dependent RNA polymerase [Bacteriophage sp.] | 0.0 | | 100 | | RNA-dependent RNA polymerase YonO [Bacillus phage Spbeta] | UniProt: O64076 | 100 | 1.9e-99 | |
| 30 | hypothetical protein | + | 12,100 | 12,822 | 723 | hypothetical protein [Bacteriophage sp.] | 9e-174 | | 99.16 | | - | - | - | - | |
| 31 | hypothetical protein | + | 12,871 | 12,972 | 102 | - | - | | - | | - | - |  |  | |
| 32 | histone-like DNA-binding protein | + | 13,042 | 13,398 | 357 | hypothetical protein CB457P1_00005 [Enterocloster phage CB457P1] | 2e-76 | | 97.46 | | histone-like DNA-binding superfamily protein [Mycoplasma gallisepticum S] | PDB: 2NDP_B | 99.84 | 1.6e-19 | |
| 33 | DNA-binding protein | + | 13,457 | 13,951 | 495 | hypothetical protein CB457P1_00006 [Enterocloster phage CB457P1] | 4e-112 | | 97.56 | | DNA-binding protein | PDB:2NP2_B | 99.77 | 2.1e-17 | |
| 34 | hypothetical protein | + | 14,017 | 14,106 | 90 | - | - | | - | | - | - |  |  | |
| 35 | hypothetical protein | + | 14,112 | 15,020 | 909 | TPA: restriction endonuclease [Caudoviricetes sp.] | 0.0 | | 99.67 | | - | - |  |  | |
| 36 | terminase, large subunit | + | 15,032 | 16,930 | 1,899 | TPA: large terminase [Caudoviricetes sp.] | 0.0 | | 99.84 | | terminase, large subunit | PDB:3CPE_A | 100 | 4.9e-33 | |
| 37 | portal protein | + | 17,030 | 18,655 | 1,626 | hypothetical protein CB457P1_00012 [Enterocloster phage CB457P1] | 0.0 | | 98.15 | | portal protein [Thermus phage P7426] | PDB: 5NGD_B | 99.31 | 4.4e-9 | |
| 38 | hypothetical protein | + | 18,688 | 18,795 | 108 | - | - | | - | | - | - | - | - | |
| 39 | ferritin | + | 18,788 | 19,288 | 501 | TPA: hypothetical protein [Caudoviricetes sp.] | 1e-117 | | 100 | | ferritin [Chlorobaculum tepidum] | PDB: 4CMY_U | 99.95 | 5.9e-26 | |
| 40 | hypothetical protein | + | 19,261 | 19,518 | 258 | hypothetical protein CB457P1_00014 [Enterocloster phage CB457P1] | 5e-47 | | 96.51 | | - | - |  |  | |
| 41 | DUF724 | + | 19,527 | 21,359 | 1,833 | TPA: Protein of unknown function (DUF724) [Caudoviricetes sp.] | 0.0 | | 98.69 | | - | - |  |  | |
| 42 | hypothetical protein | + | 21,386 | 21,934 | 549 | TPA: hypothetical protein [Caudoviricetes sp.] | 1e-126 | | 98.35 | | - | - |  |  | |
| 43 | major capsid protein | + | 21,965 | 23,068 | 1,104 | TPA: capsid protein [Caudoviricetes sp.] | 0.0 | | 99.46 | | major capsid protein Gp27 [Bacillus virus G] | PDB: 6WKK_F | 99.33 | 6.7e-10 | |
| 44 | hypothetical protein | + | 23,158 | 23,850 | 693 | hypothetical protein CB457P1_00018 [Enterocloster phage CB457P1] | 4e-163 | | 95.52 | | - | - |  |  | |
| 45 | hypothetical protein | + | 23,874 | 24,323 | 450 | hypothetical protein [Bacteriophage sp.] | 8e-96 | | 89.93 | | - | - |  |  | |
| 46 | hypothetical protein | + | 24,323 | 24,565 | 243 | hypothetical protein CB457P1_00020 [Enterocloster phage CB457P1] | 1e-45 | | 97.50 | | - | - |  |  | |
| 47 | hypothetical protein | + | 24,581 | 25,852 | 1,272 | TPA: head closure knob [Caudoviricetes sp.] | 0.0 | | 99.53 | | - | - |  |  | |
| 48 | hypothetical protein | + | 25,864 | 26,433 | 570 | TPA: hypothetical protein [Caudoviricetes sp.] | 2e-136 | | 99.47 | | - | - |  |  | |
| 49 | hypothetical protein | + | 26,426 | 27,226 | 801 | TPA: hypothetical protein [Caudoviricetes sp.] | 0.0 | | 99.62 | | - | - |  |  | |
| 50 | major tail protein | + | 27,255 | 28,301 | 1,047 | transmembrane protein [Bacteriophage sp.] | 0.0 | | 99.43 | | major tail protein [Bacteriophage sp.] | PDB: 6XGR_A | 99.67 | 7.0e-13 | |
| 51 | hypothetical protein | + | 28,389 | 28,736 | 348 | hypothetical protein [Bacteriophage sp.] | 8e-78 | | 99.12 | | - | - |  |  | |
| 52 | hypothetical protein | + | 28,802 | 29,248 | 447 | hypothetical protein CB457P1_00026 [Enterocloster phage CB457P1] | 4e-101 | | 96.62 | | - | - |  |  | |
| 53 | mRNA interferase | + | 29,280 | 29,636 | 357 | PemK-like, MazF-like toxin of type II toxin-antitoxin system [Bacteriophage sp.] | 3e-81 | | 100 | | mRNA interferase [Escherichia phage MS2] | PDB: 5DLO_A | 99.9 | 2e-21 | |
| 54 | Macoilin family protein | + | 29,669 | 30,043 | 375 | Macoilin family protein [Bacteriophage sp.] | 7e-83 | | 100 | | - | - |  |  | |
| 55 | holin | + | 30,093 | 30,359 | 267 | holin [Bacteriophage sp.] | 1e-54 | | 100 | | holin [Listeria phage A500] | UniProt: Q37977 | 99.79 | 6.2e-18 | |
| 56 | hypothetical protein | + | 30,405 | 30,503 | 99 | - | - | | - | | - | - |  |  | |
| 57 | hypothetical protein | + | 30,484 | 30,915 | 432 | hypothetical protein [Bacteriophage sp.] | 1e-95 | | 100 | | - | - |  |  | |
| 58 | hypothetical protein | + | 30,925 | 31,437 | 513 | hypothetical protein [Bacteriophage sp.] | 1e-123 | | 100 | | - | - |  |  | |
| 59 | RuvC endonuclease | + | 31,444 | 32,019 | 576 | hypothetical protein [Bacteriophage sp.] | 5e-137 | | 100 | | RuvC endonuclease [Lactococcus virus bIL67] | PDB: 4KTW_B | 99.93 | 5.1e-24 | |
| 60 | tape measure protein | + | 32,035 | 36,561 | 4,527 | TPA: minor tail protein [Caudoviricetes sp.] | 0.0 | | 78.40 | | tape measure protein [Bacillus phage Spbeta] | UniProt: O64046 | 100 | 1.8e-26 | |
| 61 | hypothetical protein | + | 36,596 | 37,105 | 510 | hypothetical protein CB473P1_00034 [Enterocloster phage CB473P1] | 2e-55 | | 98.89 | | - | - |  |  | |
| 62 | hypothetical protein | - | 37,106 | 37,429 | 324 | hypothetical protein [Enterocloster aldenensis] | 8e-72 | | 100 | | - | - |  |  | |
| 63 | hypothetical protein | + | 37,410 | 37,733 | 324 | hypothetical protein [Bacteriophage sp.] | 2e-64 | | 95 | | - | - |  |  | |
| 64 | baseplate hub protein | + | 37,760 | 38,905 | 1,146 | TPA: hypothetical protein [Caudoviricetes sp.] | 0.0 | | 99.74 | | probable baseplate hub protein [Escherichia phage T5] | UniProt: Q6QGE9 | 97.96 | 0.00045 | |
| 65 | hypothetical protein | + | 38,898 | 39,827 | 930 | TPA: hypothetical protein [Caudoviricetes sp.] | 0.0 | | 99.68 | | - | - |  |  | |
| 66 | baseplate hub protein | + | 39,846 | 41,261 | 1,416 | hypothetical protein CB473P3_00039 [Enterocloster phage CB473P3] | 0.0 | | 100 | | probable baseplate hub protein | Uniprot: P10312 | 99.9 | 7.8e-21 | |
| 67 | Chitin-binding domain type 3 | + | 41,273 | 42,019 | 747 | TPA: hypothetical protein [Caudoviricetes sp.] | 3e-178 | | 99.19 | | Chitin-binding domain type 3 | Uniprot: SM00495 | 97.08 | 0.003 | |
| 68 | hypothetical protein | + | 42,023 | 44,860 | 2,838 | TPA: hypothetical protein [Caudoviricetes sp.] | 0.0 | | 98.84 | | - | - |  |  | |
| 69 | hypothetical protein | + | 44,900 | 45,265 | 366 | hypothetical protein CB457P1_00042 [Enterocloster phage CB457P1] | 3e-77 | | 94.21 | | - | - |  |  | |
| 70 | glycine rich protein | + | 45,296 | 46,537 | 1,242 | TPA: glycine rich protein [Caudoviricetes sp.] | 0.0 | | 98.79 | | - | - |  |  | |
| 71 | hypothetical protein | + | 46,578 | 46,862 | 285 | hypothetical protein [Bacteriophage sp.] | 2e-57 | | 93.62 | | - | - |  |  | |
| 72 | hypothetical protein | + | 46,837 | 46,959 | 123 | - | - | | - | | - | - |  |  | |
| 73 | ChiA1-BD-binding domain protein | + | 46,998 | 47,555 | 558 | TPA: ChiA1-BD-binding domain protein [Caudoviricetes sp.] | 5e-133 | | 99.46 | | - | - |  |  | |
| 74 | hypothetical protein | + | 47,660 | 47,821 | 162 | hypothetical protein CB457P1_00046 [Enterocloster phage CB457P1] | 8e-24 | | 84.91 | | - | - |  |  | |
| 75 | hypothetical protein | - | 47,770 | 47,967 | 198 | - | - | | - | | - | - |  |  | |
| 76 | hypothetical protein | + | 48,032 | 48,757 | 726 | TPA: hypothetical protein [Caudoviricetes sp.] | 2e-168 | | 96.68 | | - | - |  |  | |
| 77 | lysin | + | 48,776 | 49,696 | 921 | protein of unknown function DUF1287 [Bacteriophage sp.] | 0.0 | | 97.39 | | lysin [Pneumococcus phage Dp-1] | UniProt: O03979 | 99.65 | 7.5e-13 | |
| 78 | hypothetical protein | - | 49,720 | 49,860 | 141 | - | - | | - | | - | - |  |  | |
| 79 | hypothetical protein | + | 49,904 | 50,488 | 585 | TPA: hypothetical protein [Caudoviricetes sp.] | 5e-136 | | 99.48 | | - | - |  |  | |
| 80 | hypothetical protein | + | 50,488 | 52,041 | 1,554 | hypothetical protein CB457P2_00049 [Enterocloster phage CB457P2] | 1e-126 | | 45.17 | | - | - |  |  | |
| 81 | hypothetical protein | - | 52,094 | 52,327 | 234 | hypothetical protein CB457P2_00050 [Enterocloster phage CB457P2] | 1e-45 | | 100 | | - | - |  |  | |
| 82 | hypothetical protein | - | 52,339 | 52,782 | 444 | hypothetical protein CB457P2_00051 [Enterocloster phage CB457P2] | 1e-104 | | 100 | | - | - |  |  | |
| 83 | dUTPase | - | 52,797 | 53,441 | 645 | TPA: dUTPase [Caudoviricetes sp.] | 4e-154 | | 98.13 | | dUTPase [African swine fever virus] | PDB: 6KZ6_C | 99.93 | 4.1e-24 | |
| 84 | hypothetical protein | - | 53,474 | 53,581 | 108 | TPA: hypothetical protein [Caudoviricetes sp.] | 4e-14 | | 97.14 | | - | - |  |  | |
| 85 | hypothetical protein | - | 53,660 | 53,905 | 246 | TPA: hypothetical protein [Caudoviricetes sp.] | 4e-46 | | 95.06 | | - | - |  |  | |
| 86 | hypothetical protein | - | 53,889 | 54,188 | 300 | TPA: hypothetical protein [Caudoviricetes sp.] | 2e-53 | | 87.10 | | - | - |  |  | |
| 87 | ribonucleotide reductase (anaerobic) | - | 54,214 | 54,747 | 534 | TPA: anaerobic ribonucleoside-triphosphate reductase activating protein [Caudoviricetes sp.] | 2e-125 | | 97.74 | | anaerobic ribonucleoside-triphosphate reductase-activating protein [Enterobacteria phage T4] | UniProt: P07075 | 99.7 | 2.3e-15 | |
| 88 | ribonucleotide reductase of class III (anaerobic), large subunit (EC 1.17.4.2) | - | 54,889 | 57,105 | 2,217 | TPA: anaerobic ribonucleoside triphosphate reductase [Caudoviricetes sp.] | 0.0 | | 99.46 | | anaerobic ribonucleotide reductase-triphosphate reductase [Tequatrovirus T4] | PDB: 1H7B_A | 100 | 3.5e-65 | |
| 89 | hypothetical protein | - | 57,141 | 57,512 | 372 | TPA: hypothetical protein [Caudoviricetes sp.] | 1e-81 | | 99.19 | | - | - |  |  | |
| 90 | hypothetical protein | - | 57,509 | 57,619 | 111 | - | - | | - | | - | - |  |  | |
| 91 | hypothetical protein | - | 57,725 | 58,030 | 306 | hypothetical protein CB473P1_00057 [Enterocloster phage CB473P1] | 3e-64 | | 98 | | - | - |  |  | |
| 92 | exodeoxyribonuclease V | - | 58,015 | 61,341 | 3,327 | hypothetical protein CB457P2_00058 [Enterocloster phage CB457P2] | 0.0 | | 88.89 | | exodeoxyribonuclease V [Deinococcus radiodurans] | PDB: 3E1S_A | 100 | 9.6e-55 | |
| 93 | RNA polymerase sigma factor SigK | - | 61,341 | 61,985 | 645 | hypothetical protein CB473P1_00059 [Enterocloster phage CB473P1] | 2e-144 | | 92.06 | | RNA polymerase sigma factor SigK [Mycobacterium tuberculosis] | PDB: 4NQW_A | 99.91 | 3.4e-22 | |
| 94 | hypothetical protein | - | 62,121 | 62,306 | 186 | hypothetical protein [Bacteriophage sp.] | 2e-35 | | 96.72 | | - | - |  |  | |
| 95 | DNA polymerase III alpha subunit | - | 62,307 | 66,344 | 4,038 | DNA polymerase III alpha NTPase domain [Bacteriophage sp.] | 0.0 | | 90.34 | | DNA polymerase III alpha subunit [Thermus aquaticus] | PDB: 2HPI_A | 100 | 2e-145 | |
| 96 | hypothetical protein | - | 66,345 | 66,680 | 336 | hypothetical protein CB457P2_00066 [Enterocloster phage CB457P2] | 1e-72 | | 99.10 | | - | - |  |  | |
| 97 | HNH homing endonuclease | - | 66,684 | 67,190 | 507 | TPA: homing endonuclease [Caudoviricetes sp.] | 3e-120 | | 98.81 | | HNH homing endonuclease [Okubovirus SPO1] | PDB: 1U3E_M | 98.91 | 7.5e-9 | |
| 98 | single-stranded-DNA-specific exonuclease | - | 67,282 | 69,102 | 1,821 | TPA: single-stranded-DNA-specific exonuclease RecJ [Caudoviricetes sp.] | 0.0 | | 98.17 | | putative single-strand DNA-specific exonuclease yorK [Bacillus phage Spbeta] | UniProt: O64145 | 100 | 2.7e-74 | |
| 99 | single-stranded binding protein | - | 69,166 | 69,945 | 780 | TPA: single-stranded DNA-binding protein [Caudoviricetes sp.] | 0.0 | | 98.46 | | single-strand binding protein | PDB: 1SE8_A | 99.95 | 2.2e-24 | |
| 100 | hypothetical protein | - | 70,016 | 70,213 | 198 | hypothetical protein CB457P1_00068 [Enterocloster phage CB457P1] | 9e-38 | | 98.46 | | - | - |  |  | |
| 101 | metallopeptidase Zymogen | - | 70,206 | 70,898 | 693 | TPA: SprT-like domain-containing protein Spartan repair protease, DNA binding [Caudoviricetes sp.] | 1e-165 | | 97.83 | | metallopeptidase Zymogen [Methanocaldococcus jannaschii] | PDB: 4JIX_A | 99.03 | 7.7e-9 | |
| 102 | hypothetical protein | - | 70,919 | 71,455 | 537 | TPA: hypothetical protein [Caudoviricetes sp.] | 3e-96 | | 87.08 | | - | - |  |  | |
| 103 | hypothetical protein | - | 71,468 | 71,623 | 156 | hypothetical protein [Bacteriophage sp.] | 3e-27 | | 100 | | - | - |  |  | |
| 104 | uncharacterized protein in GP2-GP6 intergenic region | - | 71,630 | 71,908 | 279 | hypothetical protein [Bacteriophage sp.] | 3e-56 | | 98.91 | | uncharacterized 10.3 kDa protein in GP2-GP6 intergenic region [Bacillus phage SPP1] | UniProt: Q38441 | 97.05 | 0.00095 | |
| 105 | hypothetical protein | - | 71,895 | 72,176 | 282 | hypothetical protein CB457P1_00070 [Enterocloster phage CB457P1] | 1e-46 | | 95.24 | | - | - |  |  | |
| 106 | DNA primase | - | 72,118 | 73,311 | 1,194 | TPA: DNA primase [Caudoviricetes sp.] | 0.0 | | 99.75 | | DNA primase | PDB: 2AU3_A | 99.98 | 6.1e-30 | |
| 107 | hypothetical protein | + | 73,506 | 73,613 | 108 | - | - | | - | | - | - |  |  | |
| 108 | hypothetical protein | - | 73,575 | 73,667 | 93 | hypothetical protein CB457P1_00072 [Enterocloster phage CB457P1] | 3e-07 | | 35.62 | | - | - |  |  | |
| 109 | uncharacterized protein in GpA 5'region | - | 73,754 | 74,032 | 279 | hypothetical protein CB457P1_00073 [Enterocloster phage CB457P1] | 2e-56 | | 95.65 | | uncharacterized 10.2 kDa protein in GpA 5'region [Escherichia phage P2] | UniProt: Q06425 | 99.87 | 7.8e-21 | |
| 110 | host-nuclease inhibitor protein Gam | - | 74,046 | 74,537 | 492 | hypothetical protein CB457P1_00074 [Enterocloster phage CB457P1] | 3e-104 | | 94.48 | | host-nuclease inhibitor protein Gam [Desulfovibrio vulgaris] | PDB: 2P2U_A | 99.44 | 4.8e-12 | |
| 111 | hypothetical protein | - | 74,542 | 74,796 | 255 | hypothetical protein CB457P1_00075 [Enterocloster phage CB457P1] | 3e-46 | | 97.40 | | - | - |  |  | |
| 112 | DNAB-like replicative helicase | - | 74,789 | 76,294 | 1,506 | hypothetical protein CB457P2_00081 [Enterocloster phage CB457P2] | 0.0 | | 97.41 | | DNAB-like replicative helicase; ATPase | PDB: 3BGW_C | 100 | 5.8e-40 | |
| 113 | hypothetical protein | - | 76,294 | 76,956 | 663 | hypothetical protein [Bacteriophage sp.] | 1e-131 | | 82.43 | | - | - |  |  | |
| 114 | hypothetical protein | - | 77,014 | 77,865 | 852 | TPA: hypothetical protein [Caudoviricetes sp.] | 6e-179 | | 99.65 | | - | - | - | - | |
| 115 | hypothetical protein | - | 78,440 | 78,778 | 339 | hypothetical protein CB457P1_00078 [Enterocloster phage CB457P1] | 1e-72 | | 96.43 | | - | - | - | - | |
| 116 | RecA | - | 78,778 | 79,821 | 1,044 | hypothetical protein CB457P1_00079 [Enterocloster phage CB457P1] | 0.0 | | 99.14 | | protein RecA | PDB: 4PPF_A | 99.08 | 8.8e-9 | |
| 117 | hypothetical protein | - | 79,974 | 80,999 | 1,026 | TPA: hypothetical protein [Caudoviricetes sp.] | 0.0 | | 99.41 | | - | - |  |  | |
| 118 | hypothetical protein | - | 81,040 | 81,324 | 285 | hypothetical protein CB457P1_00081 [Enterocloster phage CB457P1] | 9e-59 | | 98.94 | | - | - | - | - | |
| 119 | phosphocarrier protein HPR | - | 81,385 | 81,621 | 237 | hypothetical protein CB457P1_00082 [Enterocloster phage CB457P1] | 1e-49 | | 100 | | phosphocarrier protein HPR | PDB: 1KKM_J | 99.54 | 7.9e-13 | |
| 120 | spore cortex-lytic enzyme precursor | - | 81,634 | 82,080 | 447 | hypothetical protein CB457P1_00083 [Enterocloster phage CB457P1] | 2e-98 | | 95.27 | | spore cortex-lytic enzyme; Hydrolase | PDB: 4F55_A | 99.95 | 8.1e-26 | |
| 121 | hypothetical protein | - | 82,124 | 82,324 | 201 | hypothetical protein CB457P1_00084 [Enterocloster phage CB457P1] | 9e-39 | | 89.93 | | - | - | - | - | |
| 122 | site-specific recombinase Xerd | - | 82,333 | 83,151 | 819 | hypothetical protein CB457P1_00085 [Enterocloster phage CB457P1] | 0.0 | | 98.53 | | site-specific recombinase Xerd [E. coli] | PDB: 1A0P_A | 100 | 1e-33 | |
| 123 | hypothetical protein | - | 83,240 | 83,560 | 321 | hypothetical protein CB457P1_00086 [Enterocloster phage CB457P1] | 1e-66 | | 93.40 | | - | - |  |  | |
| 124 | hypothetical protein | - | 83,570 | 83,998 | 429 | hypothetical protein CB457P1_00087 [Enterocloster phage CB457P1] | 5e-96 | | 96.48 | | - | - |  |  | |
| 125 | hypothetical protein | + | 84,209 | 84,364 | 156 | - | - | | - | | - | - |  |  | |
| 126 | methyltransferase Dmt | - | 84,314 | 84,841 | 528 | hypothetical protein [Enterocloster clostridioformis] | 1e-112 | | 94.61 | | methyltransferase Dmt [Escherichia phage P1] | UniProt: Q71TL0 | 99.85 | 7.6e-21 | |
| 127 | uncharacterized protein in denB-rIIB intergenic region | - | 84,911 | 85,186 | 276 | hypothetical protein [Enterocloster] | 1e-56 | | 92.31 | | uncharacterized 7.5 kDa protein in denB-rIIB intergenic region [Enterobacteria phage T4] | UniProt: P39251 | 99.73 | 6.4e-17 | |
| 128 | hypothetical protein | - | 85,218 | 85,340 | 123 | hypothetical protein [Enterocloster aldenensis] | 9e-17 | | 85.00 | | - | - | - | - | |
| 129 | hypothetical protein | + | 85,403 | 85,567 | 165 | - | - | | - | | - | - | - | - | |
| 130 | restriction alleviation protein Lar | - | 85,553 | 85,696 | 144 | restriction alleviation protein Lar [Bacteriophage sp.] | 6e-24 | | 100 | | - | - | - | - | |
| 131 | hypothetical protein | - | 85,684 | 85,785 | 102 | - | - | | - | | - | - | - | - | |
| 132 | hypothetical protein | - | 85,831 | 86,418 | 588 | hypothetical protein CB457P1_00088 [Enterocloster phage CB457P1] | 4e-101 | | 86.67 | | - | - | - | - | |
| 133 | hypothetical protein | - | 86,481 | 86,762 | 282 | hypothetical protein CB457P1_00089 [Enterocloster phage CB457P1] | 3e-56 | | 93.75 | | - | - | - | - | |
| 134 | hypothetical protein | - | 86,777 | 86,953 | 177 | hypothetical protein [Murimonas intestini] | 6e-23 | | 70.18 | | - | - | - | - | |
| 135 | hypothetical protein | - | 86,934 | 87,131 | 198 | - | - | | - | | - | - | - | - | |
| 136 | hypothetical protein | + | 87,148 | 87,267 | 120 | - | - | | - | | - | - | - | - | |
| 137 | hypothetical protein | + | 87,288 | 87,452 | 165 | - | - | | - | | - | - | - | - | |
| 138 | hypothetical protein | + | 87,536 | 87,625 | 90 | - | - | | - | | - | - | - | - | |
| 139 | hypothetical protein | + | 87,703 | 87,855 | 153 | - | - | | - | | - | - | - | - | |
| 140 | DUF1425 | - | 88,10 | 88,612 | 513 | hypothetical protein CB457P2_00099 [Enterocloster phage CB457P2] | 4e-92 | | 78.61 | | DUF1425 family member | PDB: 3O0L_B | 98.07 | 0.00023 | |
| 141 | hypothetical protein | - | 88,636 | 89,250 | 615 | hypothetical protein CB457P2_00100 [Enterocloster phage CB457P2] | 8e-93 | | 65.89 | | - | - | - | - | |
| 142 | DUF262 domain-containing protein | - | 89,386 | 90,813 | 1,428 | DNA-sulfur modification-associated protein [Bacteriophage sp.] | 0.0 | | 88.77 | | DUF262 domain-containing protein | PDB: 7P9K_B | 99.79 | 7.7e-18 | |
| 143 | hypothetical protein | - | 90,989 | 91,087 | 99 | - | - | | - | | - | - | - | - | |
| 144 | hypothetical protein | - | 91,098 | 91,451 | 354 | hypothetical protein CB473P1_00103 [Enterocloster phage CB473P1] | 3e-19 | | 44.25 | | - | - | - | - | |
| 145 | hypothetical protein | - | 91,475 | 91,672 | 198 | hypothetical protein CB457P1_00095 [Enterocloster phage CB457P1] | 3e-30 | | 87.50 | | - | - | - | - | |
| 146 | hypothetical protein | - | 91,687 | 92,427 | 741 | hypothetical protein [Bacteriophage sp.] | 2e-157 | | 84.96 | | - | - | - | - | |
| 147 | hypothetical protein | - | 92,518 | 92,607 | 90 | - | - | | - | | - | - | - | - | |
| 148 | hypothetical protein | - | 92,760 | 92,951 | 192 | hypothetical protein CB457P2_00108 [Enterocloster phage CB457P2] | 1e-32 | | 91.80 | | - | - | - | - | |
| 149 | hypothetical protein | - | 93,022 | 93,210 | 189 | hypothetical protein CB457P2_00109 [Enterocloster phage CB457P2] | 3e-33 | | 91.94 | | - | - | - | - | |
| 150 | hypothetical protein | - | 93,239 | 93,445 | 207 | - | - | | - | | - | - | - | - | |
| 151 | hypothetical protein | - | 93,461 | 93,553 | 93 | - | - | | - | | - | - | - | - | |
| 152 | hypothetical protein | + | 93,667 | 93,864 | 198 | - | - | | - | | - | - | - | - | |
| 153 | hypothetical protein | + | 93,971 | 94,078 | 108 | - | - | | - | | - | - | - | - | |
| 154 | hypothetical protein | + | 94,149 | 94,289 | 141 | - | - | | - | | - | - | - | - | |
| 155 | hypothetical protein | - | 94,273 | 94,560 | 288 | hypothetical protein [Bacteriophage sp.] | 7e-56 | | 86.32 | | - | - | - | - | |
| 156 | hypothetical protein | - | 94,564 | 94,893 | 330 | hypothetical protein [Bacteriophage sp.] | 1e-68 | | 95.41 | | - | - | - | - | |
| 157 | hypothetical protein | - | 94,917 | 95,099 | 183 | hypothetical protein [Bacteriophage sp.] | 7e-33 | | 93.33 | | - | - | - | - | |
| 158 | hypothetical protein | - | 95,090 | 95,458 | 369 | hypothetical protein CB473P1_00113 [Enterocloster phage CB473P1] | 9e-62 | | 75.40 | | - | - | - | - | |
| 159 | hypothetical protein | - | 95,463 | 95,648 | 186 | TPA: hypothetical protein [Oscillospiraceae bacterium] | 6e-14 | | 55.93 | | - | - | - | - | |
| 160 | hypothetical protein | - | 95,659 | 96,093 | 435 | hypothetical protein CB457P2_00114 [Enterocloster phage CB457P2] | 2e-83 | | 81.25 | | - | - | - | - | |
| 161 | hypothetical protein | - | 96,097 | 96,546 | 450 | hypothetical protein CB473P3_00120 [Enterocloster phage CB473P3] | 2e-62 | | 66.44 | | - | - | - | - | |
| 162 | hypothetical protein | - | 96,559 | 97,188 | 630 | hypothetical protein CB473P3_00121 [Enterocloster phage CB473P3] | 3e-110 | | 75.47 | | - | - | - | - | |
| 163 | hypothetical protein | - | 97,201 | 97,494 | 294 | hypothetical protein CB473P3_00122 [Enterocloster phage CB473P3] | 3e-57 | | 86.60 | | - | - | - | - | |
| 164 | hypothetical protein | - | 97,497 | 97,904 | 408 | hypothetical protein CB473P1_00117 [Enterocloster phage CB473P1] | 2e-53 | | 62.12 | | - | - | - | - | |
| 165 | hypothetical protein | - | 97,986 | 98,189 | 204 | - | - | | - | | - | - | - | - | |
| 166 | hypothetical protein | + | 98,261 | 98,371 | 111 | - | - | | - | | - | - | - | - | |
| 167 | hypothetical protein | + | 98,343 | 98,441 | 99 | - | - | | - | | - | - | - | - | |
| 168 | hypothetical protein | + | 98,585 | 98,680 | 96 | - | - | | - | | - | - | - | - | |
| 169 | hypothetical protein | + | 98,665 | 98,766 | 102 | - | - | | - | | - | - | - | - | |
| 170 | hypothetical protein | + | 98,863 | 98,976 | 114 | - | - | | - | | - | - | - | - | |
| 171 | hypothetical protein | + | 99,011 | 99,121 | 111 | - | - | | - | | - | - | - | - | |
| 172 | hypothetical protein | + | 99,197 | 99,355 | 159 | - | - | | - | | - | - | - | - | |
| 173 | hypothetical protein | - | 99,441 | 99,596 | 156 | - | - | | - | | - | - | - | - | |
|  |  |  |  |  |  |  |  | |  | |  |  |  |  | |
