# Supplementary material for: Complete genome sequence of the novel virulent phage PMBT24 infecting Enterocloster bolteae from the human gut
Source: Heliyon. 2024 Apr 5;10(8):e28813. doi: 10.1016/j.heliyon.2024.e28813 (PMC11035940; doi:10.1016/j.heliyon.2024.e28813)
Supplement: Multimedia component 3 [file mmc3.docx]

**Supplementary Table S3**. Predicted host and phage promoter sequences (scores ≤ 0.9) in the genome of phage PMBT24 as determined by PhagePromoter.

| **Promoter** | **Strand** | **Position nt** | **Promoter Sequence** | **Type** | **Scores** |
| --- | --- | --- | --- | --- | --- |
| 1 | + | 1012..1041 | GTTTAGTCATACAATTTCTATTGCTAAAAT | host | 0.927 |
| 2 | + | 1524..1531 | TATAAATA | host | 0.901 |
| 3 | + | 1629..1655 | TTGACATGTGATTATATACAATAAAAT | host | 0.992 |
| 4 | + | 2098..2123 | TTGACAGTGTAAATGATATCTACAAT | host | 0.973 |
| 5 | + | 6954..6961 | AATAAATA | host | 0.908 |
| 6 | + | 7349..7377 | TTGACATATGAAAATGTATATGATATCAT | host | 0.996 |
| 7 | + | 9021..9028 | AATAAATA | host | 0.906 |
| 8 | + | 9066..9094 | TTGACTTTTATTATCTCTTATAGTAAAAT | host | 0.958 |
| 9 | + | 10627..10655 | TTGACAAAATAAAATAAATGCTTTATACT | host | 0.917 |
| 10 | + | 11335..11363 | TAAAAAATGAAAAAATTAATTGCTAAAAT | host | 0.907 |
| 11 | + | 11910..11938 | TTGACAGATTCATAAAATGATGATATAAT | host | 0.997 |
| 12 | + | 12061..12089 | TTGACTTTCACATGGAATCATGGTAAAAT | host | 0.996 |
| 13 | + | 12115..12122 | AATAAATA | host | 0.915 |
| 14 | + | 12140..12147 | TATAAATA | host | 0.960 |
| 15 | - | 12196..12203 | TATAAATA | host | 0.919 |
| 16 | + | 12901..12929 | TTTACAATTTTTTATTTTTATGTTATAAT | host | 0.994 |
| 17 | - | 13627..13634 | AATAAATA | host | 0.939 |
| 18 | - | 13993..14021 | GTTTATAACCAGCAATAAATTTATATAAT | host | 0.913 |
| 19 | + | 14074..14081 | TATAAATA | host | 0.985 |
| 20 | + | 14168..14196 | TTGACTTATCATAAAGTATGTGATATACT | host | 0.975 |
| 21 | - | 14550..14578 | TTGAATAGTTACTTTAAATCTGTTATAAT | host | 0.994 |
| 22 | + | 16196..16223 | TTGATATGATGGCTGACTATTTTAAAAT | host | 0.948 |
| 23 | + | 16771..16778 | TATAAATA | host | 0.978 |
| 24 | - | 17723..17751 | TTGACATTTATGAATACCTTCCATAAAAT | host | 0.981 |
| 25 | - | 19141..19168 | TTTGCTTTATCTCCTTTTCTGTTATATT | host | 0.984 |
| 26 | - | 21542..21569 | TTGACAAGTTACATTTTATAAAAATAAT | host | 0.908 |
| 27 | - | 21911..21933 | ATATATACCCCACTATAAGGACG | phage | 0.972 |
| 28 | + | 22947..22975 | TTGACAAAAGATAAATGAAATGATATAAT | host | 1.000 |
| 29 | + | 23632..23660 | AGAAATAAAGAAATGGGAGGATTTATAAT | host | 0.917 |
| 30 | + | 24080..24107 | TTGATATAAAGAAAATGTTGGCTAAAAT | host | 0.949 |
| 31 | - | 24605..24631 | TATCCTTTAACTTTGGCGTGGTAAAAT | host | 0.984 |
| 32 | + | 25156..25182 | AATGAATAAAATTGAAGTATATAAAAT | host | 0.930 |
| 33 | + | 25369..25398 | TGGAATGATATATAACAAAAATAATATAAT | host | 0.971 |
| 34 | + | 26566..26596 | TTGACAAAAAGTATTTTATATGATATATTAT | host | 0.940 |
| 35 | + | 26696..26721 | ATTGCTTTAGTATTAGGGGCTACAAT | host | 0.962 |
| 36 | + | 27549..27577 | TTGACAAACAGTACGAAAAAATGTATGAT | host | 0.986 |
| 37 | + | 28129..28156 | AATGCTTTTAAACGTAAAGTGTTATATT | host | 0.979 |
| 38 | + | 29002..29030 | TTGACTTCTAAAGATAAGTATGATATAAT | host | 1.000 |
| 39 | + | 29297..29304 | AATAAATA | host | 0.981 |
| 40 | + | 31285..31311 | TTTAAATTTTTTAAATGTTAATATAAT | host | 0.902 |
| 41 | - | 31371..31399 | TTGATTGCTGATTATAACTGTTTTATAAT | host | 0.944 |
| 42 | + | 32668..32696 | TTGACAATTATATGCAATTATGTTATAAT | host | 1.000 |
| 43 | - | 32698..32705 | TATAAATA | host | 0.984 |
| 44 | + | 32702..32709 | TATAAATA | host | 0.962 |
| 45 | + | 33625..33653 | TTGATAATCATAATCATTCATGTTATAGT | host | 0.954 |
| 46 | - | 37485..37511 | TTCACTTTTACGATATCCTGATATCAT | host | 0.915 |
| 47 | - | 37665..37693 | TTGACACAACTTTCTGATGGAAATAAAAT | host | 0.968 |
| 48 | + | 37906..37934 | TTGACTTTTATATATAAATATGTTATTAT | host | 0.995 |
| 49 | + | 39435..39460 | TTGAAAGTCAAAAGATTTGGTATGAT | host | 0.932 |
| 50 | + | 40023..40050 | TTGATTTAACAGACAATGAAATTAAAAT | host | 0.933 |
| 51 | - | 41086..41093 | TATAAATA | host | 0.949 |
| 52 | + | 42010..42035 | TGGACAAAAGGAAAGTATTATAAAAT | host | 0.918 |
| 53 | - | 42328..42335 | AATAAATA | host | 0.917 |
| 54 | + | 42334..42362 | TTGACAAACTATATATTGTGGTGTATAAT | host | 0.993 |
| 55 | + | 42752..42759 | AATAAATA | host | 0.956 |
| 56 | - | 45837..45863 | TTGCCAAGTAAGACGTTCACATATAAT | host | 0.968 |
| 57 | + | 46312..46340 | TTGACTAACTTCGTATTAAGTGGTAAAAT | host | 0.992 |
| 58 | + | 47606..47613 | TATAAATA | host | 0.959 |
| 59 | - | 51222..51244 | AAATACTAGTGCCTATAAAGGAA | phage | 0.908 |
| 60 | - | 51223..51245 | AAAATACTAGTGCCTATAAAGGA | phage | 0.914 |
| 61 | + | 53029..53036 | TATAAATA | host | 0.972 |
| 62 | - | 55081..55088 | TATAAATA | host | 0.983 |
| 63 | + | 55297..55304 | AATAAATA | host | 0.919 |
| 64 | - | 55544..55569 | ATTGATTATGACACATATAATATAAT | host | 0.901 |
| 65 | - | 56597..56625 | TTGAGTTTTAAAGAGTGGCATGATATTAT | host | 0.931 |
| 66 | + | 58937..58944 | TATAAATA | host | 0.930 |
| 67 | - | 60136..60167 | TTGATTGAACCATCGGTTGATACTTCTACAAT | host | 0.925 |
| 68 | - | 61528..61557 | TTGACTAATGATTGTAAAGAAGGATATATT | host | 0.964 |
| 69 | + | 61634..61664 | TTGACTGTATACCCCTTTACCTCTATATAAT | host | 0.981 |
| 70 | + | 61832..61860 | TCAAATAAAAAACTTGAAGCATTTATAAT | host | 0.936 |
| 71 | - | 63333..63340 | TATAAATA | host | 0.955 |
| 72 | - | 70475..70482 | TATAAATA | host | 0.951 |
| 73 | - | 71182..71210 | TTGACTTAATGATAATATTATGATATAAT | host | 1.000 |
| 74 | + | 73090..73118 | TTGATACATAATCAATTGAATGATATTAT | host | 0.976 |
| 75 | + | 73105..73133 | TTGAATGATATTATCAAAATCTTTATAAT | host | 0.952 |
| 76 | - | 74040..74068 | TTGCCATCCGTCCCTTATAACATTATAAT | host | 0.941 |
| 77 | + | 75244..75272 | TTGAATAGTGTATTTAGTAGTGGTATTAT | host | 0.925 |
| 78 | - | 75606..75613 | TATAAATA | host | 0.988 |
| 79 | + | 75981..76011 | TTGAAACTTCTCTTGATTAGTTTGATATAAT | host | 0.986 |
| 80 | - | 79285..79312 | TTGACAGTGTAGAGAAAGACCATATCAT | host | 0.977 |
| 81 | + | 80098..80105 | AATAAATA | host | 0.956 |
| 82 | - | 83081..83088 | AATAAATA | host | 0.954 |
| 83 | - | 85002..85027 | TTGAATCTGATTGGTTTAAGTATATT | host | 0.912 |
| 84 | - | 85045..85052 | AATAAATA | host | 0.912 |
| 85 | - | 89288..89318 | TTGACTGGCGATTATGAACAAACTTTAAAAT | host | 0.937 |
| 86 | - | 90377..90405 | TTGACAAAATTGTATATATAATATATAAT | host | 0.984 |
| 87 | + | 90546..90553 | TATAAATA | host | 0.946 |
| 88 | + | 90592..90599 | TATAAATA | host | 0.917 |
| 89 | - | 90869..90898 | GAAAACAAATATTTTTTCAATTGATATAAT | host | 0.933 |
| 90 | - | 91006..91013 | TATAAATA | host | 0.936 |
| 91 | + | 91096..91124 | TTGACATTTATATTATATTCTGTTATTAT | host | 0.986 |
| 92 | + | 91127..91149 | AAATAAAATAGACTAAAAGGAGG | phage | 0.969 |
| 93 | - | 91736..91761 | TTGCCATACTCAATCATAAATATATT | host | 0.905 |
| 94 | + | 92328..92350 | AATTAGGATTCACTAAAATGAGT | phage | 0.959 |
| 95 | - | 92854..92881 | TTAACATTAGCAATTTCTATGCTAAAAT | host | 0.914 |
| 96 | - | 93019..93026 | TATAAATA | host | 0.980 |
| 97 | + | 94498..94527 | GTTTATTTTTGTCTAGTTGTATGCTATAAT | host | 0.960 |
| 98 | + | 94941..94969 | TTGACATATTATAATTTATATGATATAGT | host | 0.995 |
| 99 | + | 94969..94976 | TATAAATA | host | 0.910 |
| 100 | + | 96585..96592 | TATAAATA | host | 0.957 |
| 101 | + | 97770..97797 | TTGATAATTTCAATTCGTAAAGTAAAAT | host | 0.963 |
| 102 | + | 98190..98197 | AATAAATA | host | 0.911 |
| 103 | + | 98691..98698 | TATAAATA | host | 0.968 |

**Supplementary Table S4.** *Rho*-independent terminators (ΔG ≤ -10 kcal mol^-1^) in the genome of phage PMBT24 predicted by ARNold. Loops are shown in red, stems in blue.

**Terminator Strand Position nt Terminator sequence ΔG kcal mol^-1^**

1 - 10219..10239 TCCCCATTGTTTAAATGGGGA -12.30

2 + 10219..10239 TCCCCATTTAAACAATGGGGA -10.90

3 + 11500..11521 AACCTTCCAGAAATGGAAGGTT -11.40

4 + 11502..11519 CCTTCCAGAAATGGAAGG  -10.80

5 + 21159..21182       TCCCTCTGCCGTTTGGTGGAGGGA -10.00

6 - 26143..26165   CTCCCTAGTTTAAATCTGGGGAG -13.00

7 + 30453..30475 AGGTACTCTGTCATGGAGTACCT -10.80

8 - 47507..47539 TACCCACCATCCTTATACATGTGATGGTGGGTA -12.20

9 + 47507..47539 TACCCACCATCACATGTATAAGGATGGTGGGTA -12.20

10 - 76472..76497 GCTGGGAGAAGTCACATTCTTCCAGC -12.30

11 - 94107..94132 CCCTCAGTTCATGTCGAAACTGAGGG -12.30

12 + 94107..94132 CCCTCAGTTTCGACATGAACTGAGGG -12.30

13 - 97491..97507       CCCCCGGTGTTCAAGCACCGGGG -17.10

14 - 98469..98495 AACTCCCCAGATTTAAACCGGGGAGTT -12.70

15 + 98471..98493       CTCCCCGGTTTAAATCTGGGGAG -14.60
  
